# Increases in local skin temperature correlate with spontaneous foot lifting and heat hyperalgesia in both incisional inflammatory models of pain

**DOI:** 10.1097/PR9.0000000000001097

**Published:** 2023-09-12

**Authors:** Ratan K. Banik, Twan Sia, Mohab M. Ibrahim, Eellan Sivanesan, Megan Uhelski, Adrian Pena, John M. Streicher, Donald A. Simone

**Affiliations:** a Department of Anesthesiology, University of Minnesota, Minneapolis, MN, USA; b Stanford University School of Medicine, Stanford, CA, USA; c Department of Anesthesiology, University of Arizona, Tucson, AZ, USA; d Department of Anesthesiology, Johns Hopkins University, Baltimore, MD, USA; e Department of Pain Medicine, University of Texas MD Anderson Cancer Center, Houston, TX, USA; f Department of Pharmacology, University of Arizona, Tucson, AZ, USA; g Department of Diagnostic and Biological Sciences, School of Dentistry, University of Minnesota, Minneapolis, MN, USA

**Keywords:** Hyperalgesia, Behavior, Pain

## Abstract

**Background:**

This study investigated if a localized increase in skin temperature in rat models of incisional and inflammatory pain correlates with the intensity of spontaneous and evoked pain behaviors.

**Methods:**

Anesthetized rats received either a 20-mm longitudinal incision made through the skin, fascia, and muscle of the plantar hind paw or an injection of complete Freund adjuvant into the plantar hind paw of anesthetized rats to induce local inflammation. Spontaneous and evoked pain behaviors were assessed, and changes in skin temperature were measured using a noncontact infrared thermometer.

**Results:**

There were no differences in skin temperature between the ipsilateral and contralateral hind paw before the incision or inflammation. Skin temperature increased at 2 hours after hind paw plantar incision or 1 day after inflammation of the affected paw, which gradually returned to baseline by the first day and fourth days after treatment, respectively. The increase in skin temperature correlated with the intensity of spontaneous pain behaviors and heat but not with mechanical allodynia.

**Conclusions:**

Our results suggest that a simple measurement of localized skin temperature using a noncontact infrared thermometer could measure the extent of spontaneous pain behaviors and heat hyperalgesia following plantar incision or inflammation in animals. In the absence of a reliable objective marker of pain, these results are encouraging. However, studies are warranted to validate our results using analgesics and pain-relieving interventions, such as nerve block on skin temperature changes.

## Abstract 2

Localized skin temperature increase correlated with spontaneous pain behaviors and heat hyperalgesia, but not mechanical allodynia, in rat models of incisional and inflammatory pain.

## 1. Introduction

Two cardinal features of inflammation are pain and increased tissue temperature.^28^ It is currently unknown if local temperature changes can be used as an indirect measure of the intensity of spontaneous or evoked pain. Previous studies attempting to address this question are surprisingly limited. In clinical studies, assessments of pain intensity in patients with juvenile rheumatoid arthritis correlated with increased joint temperature.^13^ After upper-extremity amputations, patients with phantom limb pain had higher stump temperatures compared with pain-free amputees.^1^ Similarly, long-term measurements of skin temperature in patients with complex regional pain syndrome showed that the affected limb had higher skin temperatures than unaffected limbs or limbs in healthy controls.^20^ In addition, a proportion of patients experiencing nummular headaches with trophic changes had increased local temperature.^23^ Furthermore, capsaicin-induced abdominal pain was associated with increased temperature in the referred pain area, whereas control subjects who were administered saline showed no such temperature changes.^2^ By contrast, a pilot study found no significant correlation between knee temperature and pain in osteoarthritis patients.^33^

Increased local skin temperature has also been documented following skin incision in humans. For example, the site of surgical wounds for enterostomy closure had elevated temperature.^30^ The wound site temperature in obese women were found to be higher than their abdominal temperature following a cesarean section.^10^ However, the animal studies showed disparate results. Woo et al.^35^ measured hind paw skin temperature using a contact wire thermocouple between operated rats and sham-operated rats, finding no significant difference in skin temperature. Similarly, in mice, Scherer et al. used infrared thermography and found increased hind paw temperatures at the site of injury for inflammatory models but not following skin incision.^29^ The reason for these discrepancies in the results from human studies and animal studies for incisional pain are unclear but may be due to methodological differences used in animal and human studies. For example, contact-based temperature measurements used in animal studies may have lower thermal sensitivity and spatial resolution.^21^ Therefore, in this study, we measured hind paw skin temperature following skin incision or inflammation of the rats using a noncontact method and compared skin temperature with measures of spontaneous pain behaviors, mechanical allodynia, and heat hyperalgesia.

## 2. Methods

### 2.1. Animals

Adult, male, Sprague-Dawley rats (250–300 g) were purchased from Harlan (Somerville, NJ) for this study. Two or 3 rats were housed together in 43 × 21.5 × 25.5 cm polymethyl methacrylate cages and kept on a 12-hour light/dark cycle. Food and water were available ad libitum.

Rats were simultaneously randomized into the incisional pain and inflammatory pain groups without considering any other variables. Six rats were used to model incisional pain, and 10 rats were used to model inflammatory pain. No control rats were needed in this experiment because treatment was only given to the ipsilateral hind paw and the contralateral hind paw served as a control. An a priori power analysis for sample size selection was not performed. The sample size was informed by previously published studies studying hind paw temperature increases in rat models of incisional pain.^35^ There was no criteria for exclusion of animals for experimentation or analysis.

This study adhered to the proposals of the Committee for Research and Ethical Issues of the International Association for the Study of Pain and was approved by the Seton Hall University Institutional Animal Care and Use Committee.

### 2.2. Plantar incision and injection of complete Freund's adjuvant

Rats were anesthetized with isoflurane before surgical incision or subcutaneous injection. Each animal was placed in a plexiglass induction chamber containing 5% isoflurane in room air. Once the righting reflex was lost, 2% to 3% isoflurane in room air was delivered through a nose cone.

The rat model of incisional pain was performed as previously described.^8^ Briefly, a 20-mm longitudinal incision was made through the skin and fascia of the plantar aspect of the hind paw. Following skin incision, the plantaris muscle was elevated, stressed, and incised longitudinally, although the origin and insertion of the muscle remained intact. The skin was then closed using 2 mattress 5-0 silk sutures. After surgery, animals were housed on soft bedding and allowed to recover. Sutures were removed on the third postoperative day.

To produce inflammation of the hind paw, each rat received 100 μL of 1 μg/mL complete Freund adjuvant (CFA) (Sigma-Aldrich, St. Louis, MO) injected subcutaneously into one plantar hind paw using a sterile 1-mL syringe with a 27-G, 1/2-inch needle. In subsequent measurements, investigators could not be blinded to which paw was ipsilateral or contralateral.

### 2.3. Measurement of plantar skin temperature

Plantar skin temperature difference measurements were performed at 2 hours, and at 1, 2, 3, 6, and 8 days after incision or at 1, 2, 3, 4, 7, and 9 days after CFA injection. Animals were placed on metal mesh floor (IITC Life Sciences, Woodland Hills, CA) and allowed to acclimate (room temperature 22–24°C) for 30 minutes. Mid plantar skin temperature was measured bilaterally with a noncontact infrared thermometer (range 0–600°F, emissivity 0.2–1, target size 1/4-inch diameter at 3/4-inch distance; Omega Engineering, Stamford, CT). The sensor was placed directly under the paw through gaps in the metal mesh. The distance between the skin and the sensor was approximately 5 mm and was kept constant. Temperature readings were recorded on the injured and uninjured paw after 5 to 10 seconds of stabilization. The order of measurement was random because a noncontact thermometer is not known to cause any physiologic change in the recorded tissue.^21^ The difference in plantar skin temperature between the hind paws was calculated by the following formula:

Temperature difference=skin temperature on injured paw−skin temperature on uninjured paw.

### 2.4. Spontaneous foot lifting behaviors

To measure spontaneous pain behaviors, spontaneous foot lifting (SFL) was measured as previously described.^15^ Measurements were performed at 2 hours and at 1, 2, 3, 6, and 8 days after incision or at 1, 2, 3, 4, 7, and 9 days after CFA injection. Briefly, after rats were placed on a metal mesh floor and allowed to acclimate for 20 minutes. The spontaneous and rapid lifting episodes of the paw was counted with a manual counter. A mirror was placed under the mesh floor at a 45° angle to view both hind paws clearly. Spontaneous foot lifting behaviors manifested as 3 distinct patterns: (1) spontaneous, rapid, and brief lifting of the incised paw; (2) rapid lifting followed by licking; and (3) rapid lifting followed by holding the paw off the mesh for a brief period. The behaviors were quantified by a trained investigator. Paw lifting associated with locomotion were not counted. Behaviors with a duration of <1 second were ignored. Three rats were tested at one time. Rats were selected randomly each day. Each rat was observed in 4 blocks with 5-minute intervals and 10-minute rest periods over the duration of an hour. The total observation per rat was 20 minutes.

### 2.5. Measurement of evoked mechanical allodynia

Following SFL measurements, the same rats were tested for mechanical allodynia at the following time points: 2 hours and 1, 2, 3, 6, 8, 10, and 13 days after incision; and 1, 2, 3, 4, 7, 9, 11, 14, 16, 18, 25, and 32 days after CFA injection. The measurement order was selected randomly. Rats were placed under plexiglass compartments (12 × 20 × 17 cm) on a metal mesh floor. The paw withdrawal threshold (PWT), or the threshold force required for paw withdrawal in grams, was determined using an electronic von Frey consisting of a handheld force transducer attached to 0.5-mm polypropylene tips (IITC Life Sciences). After the rats had acclimated, force was applied perpendicularly from the polypropylene tip to the midplantar surface, starting with the least rigid tip. If a flinching response accompanied by spastic paw withdrawal was observed, stimulation with the same filament was repeated following reacclimation to ensure validity. If the response was reproduced, then the response was recorded. If a response was not reproduced, it was ignored and the protocol was repeated using a filament with a higher rigidity. The PWT was calculated by averaging measurements from 3 to 4 independent trials.

### 2.6. Measurement of hyperalgesia to heat

After PWT measurements, sensitivity to heat was determined by measuring the paw withdrawal latency (PWL) to noxious heat. Paw withdrawal latency was measured at 2 hours and at 1, 2, 3, 6, 8, 10, and 13 days after incision or at 2, 3, 4, 7, 9, 11, 14, 16, and 18 days after CFA injection. The measurement order was selected randomly. Animals were placed on an elevated glass floor in individual plexiglass chambers (12 × 20 × 17 cm) and allowed to acclimate for 15 minutes. Convective heat was used to maintain the temperature of the glass floor at 29.0 ± 1°C. A focused radiant heat source (50 W projector lamp with an aperture diameter of 6 mm) was positioned directly beneath the midplantar region of the ipsilateral hind paw. Stimulus temperature values began at 40°C and increased in 2.5°C increments every 10 seconds until a clear withdrawal response occurred.

For subsequent trials, baseline glass temperature (29.0 ± 1°C) and animal behavior were reestablished, and the input stimulus temperature was set at 2.5°C below the temperature associated with the PWL of the previous trial. To maintain consistent heat intensities across individual stimuli at the same input temperatures, compressed air was used to remove heat from the lamp to maintain a temperature of 29.0 ± 1°C before stimulation. No more than one trial was applied at a given location on the glass floor to avoid elevating the skin temperature. If after one trial, the animal did not move to another location, movement was elicited by gently tapping the glass floor.

### 2.7. Statistical analyses

Time-dependent changes in differences in plantar hind paw skin temperature, frequency of SFL, PWT, and PWL were analyzed using a 1-way analysis of variance (ANOVA) followed by a post hoc Dunnett test. Analysis of variance model assumptions were checked using a Shapiro–Wilk test and Bartlett test. The post hoc Dunnett test was used to compare each time point with the time point furthest from injury. For rats with plantar incision, the latest time point was day 8 for hind paw skin temperature difference and SFL and day 13 for PWT and PWL. For CFA-injected rats, the latest time point was day 9 for hind paw skin temperature difference, day 32 for PWT, day 18 for PWL, and day 9 for SFL. The latest time points were used for comparison because those measurements were similar to baseline pain behavior measurements that were previously reported in the literature.^15,34^

The primary outcome of this study was the association of hind paw skin temperature difference with frequency of SFL, PWT, or PWL. For each animal model, at each time point that both temperature and pain behavior measurements were available, the mean hind paw skin temperature difference was plotted against the mean SFL count, PWT, or PWL for that time point. A linear regression was plotted, and the strength and direction of the association was determined using the Pearson correlation coefficient. Analyses were performed using Prism 5 (GraphPad Software, San Diego, CA). A *P* value of <0.05 was considered significant.

## 3. Results

### 3.1. Relation between local skin temperature and pain behaviors following skin incision

**Figure 1.**
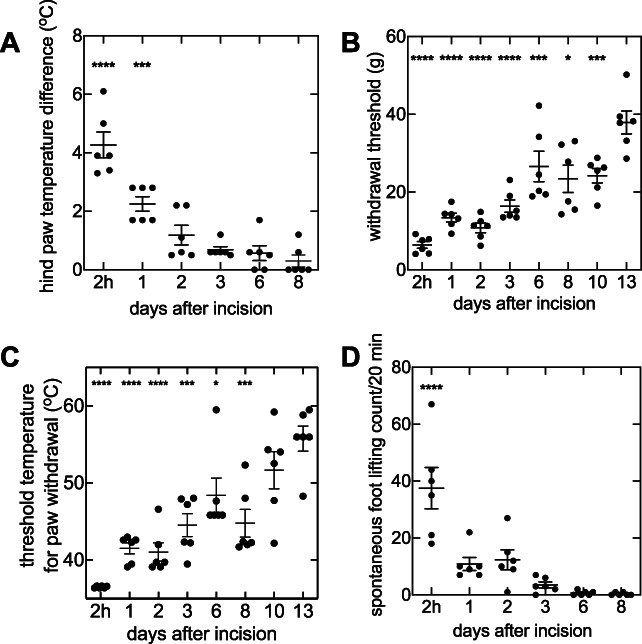
Hind paw temperature difference and pain behaviors after plantar incision in the rat hind paw (n = 6). For each measurement, a 1-way ANOVA was performed as an omnibus test, and a post hoc Dunnett test was used to compare each measurement at each time point against their respective last time point measurement (day 8 for skin temperature difference and SFL count and day 13 for PWT and PWL). (A) Hind paw temperature difference between the ipsilateral and contralateral paws as measured using a noncontact infrared thermometer. Skin temperature difference varied over the measurement period (1-way ANOVA F(5,30) = 27.94, *P* < 0.0001). Skin temperature difference was significantly higher than day 8 at 2 hours (*P* < 0.0001) and 1 day (*P* = 0.0002) after plantar incision. (B) Evoked mechanical allodynia was measured following plantar incision. PWT was found to be significantly different over the measurement period (1-way ANOVA F(7,40) = 17.94, *P* < 0.0001). When compared with day 13, PWT was found to be significantly different 2 hours (*P* < 0.0001), 1 day (*P* < 0.0001), 2 days (*P* < 0.0001), 3 days (*P* < 0.0001), 6 days (*P* = 0.0106), 8 days (*P* = 0.0007), and 10 days (*P* = 0.0014) after plantar incision. (C) Heat hyperalgesia was measured using PWL (1-way ANOVA F(7,40) = 14.78, *P* < 0.0001). PWL at 2 hours (*P* < 0.0001), 1 day (*P* < 0.0001), 2 days (*P* < 0.0001), 3 days (*P* = 0.0001), 6 days (*P* = 0.0149), and 8 days (*P* = 0.0002) were significantly different from measurements at 13 days postplantar incision. (D) Spontaneous pain behaviors were measured using SFL count (1-way ANOVA F(5,30) = 16.48, *P* < 0.0001). SFL count was greater at 2 hours following plantar incision (*P* < 0.0001). The horizontal axis is not scaled linearly with time. Horizontal lines represent mean values. Error bars represent SEM. Asterisks denote significant differences (****P < 0.0001, ***P < 0.001, *P < 0.05). ANOVA, analysis of variance; PWL, paw withdrawal latency; PWT, paw withdrawal threshold; SFL, spontaneous foot lifting.

**Figure 2.**
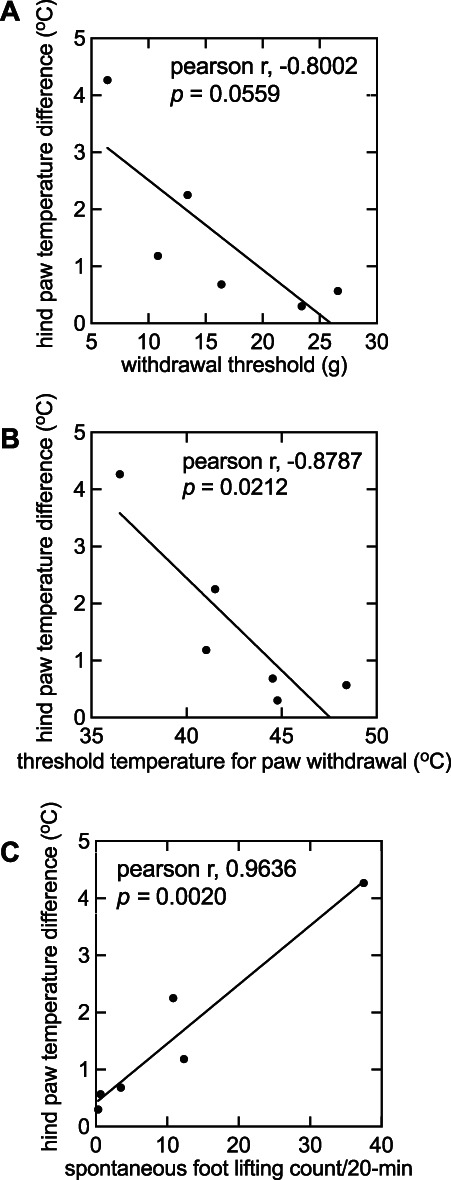
Correlation coefficients between hind paw temperature difference and various pain behaviors after plantar hind paw incision were calculated using Pearson regression analysis. (A) Evoked mechanical allodynia (*r* = −0.8002; 95% CI = −0.9772, 0.03237; *P* = 0.0559), (B) heat hyperalgesia (*r* = −0.8787; 95% CI = −0.9867, −0.2342; *P* = 0.0212), and (C) spontaneous pain behaviors (*r* = 0.9636; 95% CI = 0.6975, 0.9962; *P* = 0.0020) were analyzed.

There were no differences in temperature between the ipsilateral and contralateral hind paw before the incision (data not shown). After incision, skin temperature of the incised paw increased (1-way ANOVA, F(5,30) = 27.94, *P* < 0.0001; Fig. 1A). The maximum increase in skin temperature, 4.27 ± 0.44°C, occurred at 3 hours after incision (*P* < 0.0001) and remained elevated for 24 hours (*P* = 0.0002; Fig. 1A). Paw withdrawal threshold and PWL were measured for 13 days after skin incision, and SFL count was measured for 8 days after skin incision (Figs. 1B,D). In investigating the correlation between skin temperature and pain behaviors from 2 hours and 1, 2, 3, and 6 days after plantar incision, there was no relationship between skin temperature and PWT (*r* = −0.8002; 95% CI = −0.9772, 0.03237; *P* > 0.05; Fig. 2A). However, differences in skin temperature correlated with PWL (*r* = −0.8787; 95% CI = −0.9867, −0.2342; *P* = 0.0212; Fig. 2B), and SFL count (*r* = 0.9636; 95% CI = 0.6975, 0.9962; *P* = 0.0020; Fig. 2C).

### 3.2. Relation between local skin temperature and pain behaviors following complete Freund adjuvant injection

**Figure 3.**
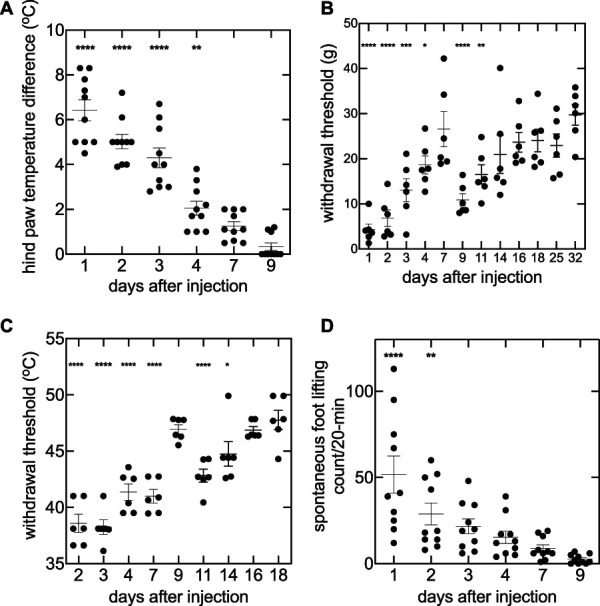
Hind paw temperature and pain behaviors after experimentally induced inflammation in the rat plantar hind paw. Intraplantar injection of complete Freund adjuvant (CFA) produces inflammation and alterations in nociceptive sensitivity. For each measurement, an omnibus test was performed using a 1-way ANOVA, and comparisons of each measurement at each time point against their respective last time point measurement (day 9 for skin temperature difference and SFL count, day 32 for PWT, day 18 for PWL, and day 9 for SFL count) was performed using a post hoc Dunnett test. (A) Hind paw temperature difference between the inflamed and noninflamed paw were measured using an infrared thermometer. Hind paw temperature differences were varied over the measurement period (n = 10; 1-way ANOVA F(5,54) = 49.47, *P* < 0.0001). In comparison to day 9, skin temperature was elevated on day 1 (*P* < 0.0001), day 2 (*P* < 0.0001), day 3 (*P* < 0.0001), and day 4 (*P* = 0032). (B) Evoked mechanical allodynia was varied during the measurement period following CFA-induced inflammation (n = 6; 1-way ANOVA F(11,60) = 9.839, *P* < 0.0001). PWT was significantly different on day 1 (*P* < 0.0001), day 2 (*P* < 0.0001), day 3 (*P* = 0.0002), day 4 (*P* = 0.0263), day 9 (*P* < 0.0001), and day 11 (*P* = 0.0050) when compared with day 32. (C) Heat hyperalgesia was found to be significantly different over the measurement period (n = 6; One-way ANOVA F(8,45) = 26.43, *P* = <0.0001). Day 2 (*P* < 0.0001), day 3 (*P* < 0.0001), day 4 (*P* < 0.0001), day 7 (*P* < 0.0001), day 11 (*P* < 0.0001), and day 14 (*P* = 0.0259) were found to be significantly different from day 18. (D) Spontaneous evoked pain behaviors were measured using SFL count, which was significantly different over the measurement period (n = 10; 1-way ANOVA F(5,54) = 9.439, *P* < 0.0001). Day 1 (*P* < 0.0001) and day 2 (*P* = 0.0085) were significantly different from day 9. The horizontal axis is not scaled linearly with time. Horizontal lines represent mean values. Error bars represent SEM. Asterisks denote significant differences (****P < 0.0001, ***P < 0.001, **P < 0.01, *P < 0.05). ANOVA, analysis of variance; CFA, complete Freund's adjuvant; PWL, paw withdrawal latency; PWT, paw withdrawal threshold; SFL, spontaneous foot lifting.

**Figure 4.**
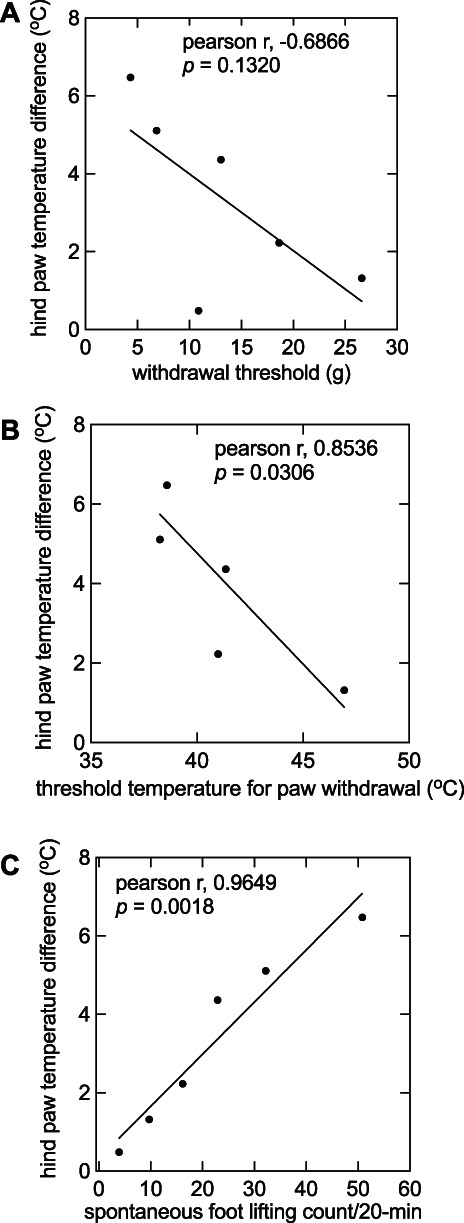
Correlation coefficients for hind paw temperature difference vs various pain behaviors were calculated with Pearson regression analysis. (A) Evoked mechanical allodynia (*r* = −0.6866; 95% CI = −0.9621, 0.2825; *P* = 0.1320), (B) heat hyperalgesia (*r* = 0.8536; 95% CI = −0.9837, −0.1367; *P* = 0.0306), and (C) evoked pain behaviors (*r* = 0.9649; 95% CI = 0.7068, 0.9963; *P* = 0.0018) were analyzed.

There were no differences in temperature between the ipsilateral and contralateral hind paw before the incision (data not shown). Intraplantar injection of CFA into the hind paw increased skin temperature as compared with the contralateral control paw (1-way ANOVA, F (5,54) = 49.47, *P* < 0.0001; Fig. 3A). The maximum difference in hind paw temperatures (6.42 ± 0.47°C) occurred on the first day. Hind paw temperature difference gradually decreased to baseline by day 4 because post hoc Dunnett test against day 9 was only significant for day 1 (*P* < 0.0001; Fig. 3A), day 2 (*P* < 0.0001; Fig. 3A), day 3 (*P* < 0.0001; Fig. 3A), and day 4 (*P* = 0.003; Fig. 3A). Paw withdrawal threshold was measured for 32 days (Fig. 3B), PWL was measured for 18 days (Fig. 3C), and SFL count was measured for 9 days after CFA injection (Fig. 3D). The correlation between changes in skin temperature and pain behaviors were examined for 1, 2, 3, 4, 7, and 9 days following CFA injection. As for skin incision, changes in skin temperature did not correlate with PWT (*r* = −0.69; 95% CI = −0.96, 0.28; *P* = 0.13; Fig. 4A) but correlated with PWL (*r* = 0.85; 95% CI = −0.98, −0.14; *P* = 0.03; Fig. 4B) and SFL count (*r* = 0.97; 95% CI = 0.71, 0.99; *P* = 0.0018; Fig. 4C).

## 4. Discussion

In this study, we characterized skin temperature difference between ipsilateral and contralateral paws in rat models of incisional and inflammatory pain and correlated changes in skin temperature with measures of spontaneous and evoked pain behaviors. Compared with the contralateral paw, skin temperature of the injured paw increased after the injury and gradually recovered over time. Although increased skin temperature after inflammation is well documented, our study is the first to show that in animal models of incisional pain, increases in local temperature at the incisional site correlated with SFL behaviors and heat hyperalgesia but not mechanical allodynia.

Our results may appear to contrast with previous work by Woo et al. (2004) and Scherer et al. (2010). However, several methodological differences may account for these apparent discrepancies between our studies. For example, Woo et al.^35^ used contact-based thermocouple to measure skin temperature, which may have lower thermal sensitivity than the noncontact infrared thermometer used in this study. In addition, Scherer et al.^29^ used infrared thermography, which may be susceptive to systemic increase in temperature, changes in ambient temperature, or other unforeseen variables. Moreover, SFL behaviors are difficult to measure in mice.^24^

Our study showed that the increased local temperature correlated well with increased intensity of spontaneous pain behaviors and heat hyperalgesia, but not evoked mechanical allodynia, after plantar incision in rats. Spontaneous foot lifting behavior in animals has been considered an expression of ongoing pain.^3,36^ Several studies suggest that the duration of spontaneous pain behaviors in our incisional model correlates well with the temporal pattern of incisional evoked pain in humans reported in previous studies.^16,17,26^ It is likely that ongoing activity in sensitized C-fiber and A-fiber nociceptors induced by deep tissue (muscle and/or fascia) injury may contribute to ongoing pain behaviors in this model. Chemical mediators that are released after tissue damage may lower the threshold for the activation of afferents so that body temperature becomes adequate stimuli for their activation.^27,31^ In agreement, our previous reports show that tissue damage by incisional injury increases the excitability of primary afferents such that they exhibit spontaneous activity and increased heat sensitivity even in an in vitro condition where blood-borne mediators are removed, and afferents are separated from the influence of their cell body and central nervous system.^4^ Several mediators, such as nerve growth factor, released after an incisional injury and tissue acidosis contribute to nociceptor sensitization^7,35^ because of their capability to reduce threshold temperature for the activation of the heat-sensitive ion channel transient receptor potential cation channel subfamily V member 1 (TRPV1).^11,14,38^ Lack of this receptor prevented the development of carrageenan, complete Freund adjuvant, or incision-induced heat hyperalgesia.^6,9,12,25^ Further research into the relationship between temperature and spontaneous pain behaviors may investigate grimacing, which is another way to measure spontaneous pain behaviors in animals.^32^

Our results show a lack of correlation between localized temperature difference and mechanical allodynia after incision and inflammation. The mechanistic basis of mechanical allodynia is incompletely understood. It has been suggested that candidate mechanosensitive channels such as Piezo2, as well as the peripheral and central nervous system mechanisms, are implicated in this form of pain.^22^ In agreement, Zahn and Brennan^37^ (1999) showed that hind paw incision produced sensitization of the dorsal horn neuron as evidenced by increases in their spontaneous activity and evoked activity. Furthermore, several single peripheral neuron recording studies showed sensitization of nociceptors to heat under many experimental conditions, but most of these studies did not show mechanical sensitization.^5^ Likely, central nervous system^18,19^ mechanisms play a larger role on this form of pain behavior.

### 4.1. Significance

Our results suggest that a simple measurement of localized skin temperature using a noncontact infrared thermometer could measure the extent of spontaneous pain behaviors and heat hyperalgesia following plantar incision or inflammation in animals. In the absence of a reliable objective marker of pain, these results are encouraging. However, studies are warranted to validate our results using analgesics and pain-relieving interventions, such as nerve block on skin temperature changes.

## Disclosures

The authors have no conflict of interest to declare.
